# Genotoxicity Assessment of Multispecies Probiotics Using Reverse Mutation, Mammalian Chromosomal Aberration, and Rodent Micronucleus Tests

**DOI:** 10.1155/2013/254239

**Published:** 2013-10-23

**Authors:** Yi-Jen Chiu, Mun-Kit Nam, Yueh-Ting Tsai, Chun-Chi Huang, Cheng-Chih Tsai

**Affiliations:** ^1^Department of Food Science and Technology, Hungkuang University, No. 1018, Sector 6, Taiwan Boulevard, Shalu District, Taichung 43302, Taiwan; ^2^New Bellus Enterprises Co. Ltd., Tainan 72042, Taiwan; ^3^Super laboratory Ltd., New Taipei 24890, Taiwan

## Abstract

Genotoxicity assessment is carried out on freeze dried powder of cultured probiotics containing *Lactobacillus rhamnosus* LCR177, *Bifidobacterium adolescentis* BA286, and *Pediococcus acidilactici* PA318. Ames tests, *in vitro* mammalian chromosome aberration assay, and micronucleus tests in mouse peripheral blood are performed. For 5 strains of *Salmonella* Typhimurium, the Ames tests show no increased reverse mutation upon exposure to the test substance. In CHO cells, the frequency of chromosome aberration does not increase in responding to the treatment of probiotics. Likewise, the frequency of micronucleated reticulocytes in probiotics-fed mice is indistinguishable from that in the negative control group. Taken together, the toxicity assessment studies suggest that the multispecies probiotic mixture does not have mutagenic effects on various organisms.

## 1. Introduction

Probiotics are microorganisms believed to confer health benefits on hosts through colonizing the gastrointestinal system, maintaining a healthy balance of microflora in guts, and thereby regulating digestion and immune functions of the host. Live probiotic bacteria can be found in fermented dairy products and probiotic fortified foods; in addition, tablets, capsules, powders, and sachets containing a complex formulation of bacteria in freeze dried form are also available. In addition to demonstrating the efficacy of probiotics in improving human health, safety characteristics must be taken into consideration. However, for new isolate-specific species or strains of probiotics, novel probiotics cannot be assumed to share the historical safety of traditional strains [1]. 

The freeze dried powder of a multispecies probiotic mixture (PROBIO S-23) includes *Lactobacillus rhamnosus* LCR177, *Bifidobacterium adolescentis* BA286, and *Pediococcus acidilactici* PA318. *L. rhamnosus* is known for its robust character to survive the acid and bile in human gastrointestinal system. It produces lactic acid, has a great avidity for human intestinal mucosal cells, and helps maintain a good balance of microflora in digestive systems [2–4]. *B. adolescentis* are the natural inhabitants in healthy humans since birth to late adulthood. The isolated species has been used to treat infant diarrhea when discovered in 1899, and its presence in the gut has been associated with a healthy microbiota [5]. More importantly, growing body of evidence has revealed more physiological functions of the species, such as relieving liver damage, preventing initiation of colon cancer, and stimulating host's immune response [6]. *Pediococcus acidilactici* is a species of lactic acid bacteria being able to colonize the digestive tract [7]. It produces bacteriocin and pediocin PA-1 and exerts antagonistic effect against other microorganisms including enteric pathogens [8]. In summary, the three species are nonpathogenic to human beings, and intakes of these probiotics are presumed to promote a beneficial gastrointestinal ecology in human bodies.

To ensure safety of multispecies probiotics consumption, the current study performs genotoxicity assessment for potential mutagenic effects derived from exposure to the multispecies probiotic mixture. The test organisms include *Salmonella* Typhimurium, mammalian tissue culture, and rodents. Our results suggest that exposure to multispecies probiotics does not provoke reverse mutation, chromosomal aberrations, and micronucleated reticulocytes in bacteria, mammalian cells, and mouse peripheral blood, respectively. Based on current tests, we conclude that multispecies probiotics do not carry mutagenicity. 

## 2. Material and Methods

### 2.1. Test Substance

Stock culture collections were maintained at −70°C in Lactobacilli MRS Broth (DIFCO, Detroit, MI, USA) containing 25% glycerol. Cells were propagated twice in Lactobacilli MRS Broth containing 0.05% L-cysteine by incubation at 37°C for 20 hours. The probiotic mixture PROBIO S-23 was manufactured by New Bellus Corporation (Tainan, Taiwan). Bacterial counts were determined by plating serial dilutions of the culture in PBS on MRS agar. Plates were incubated at 37°C for 48 h anaerobically. Multispecies probiotics contain *L. rhamnosus* LCR177, *B. adolescentis* BA286, and *P. acidilactici* PA318 with a total of 5.0 × 10^10^ CFU/g count.

### 2.2. Reverse Mutation Assay

The test bacterial strains were *Salmonella* Typhimurium TA97, TA98, TA100, TA102, and TA1535 (Bioresource Collection and Research Center, Hsinchu, Taiwan). Genotypes of these strains were confirmed by histidine requirement, rfa mutation, uvrB mutation, and ampicillin resistance before the assay. 

Plate incorporation assay was applied to detect reverse mutation [9]. In brief, 100 *μ*L water solution of test substance at 50, 25, 12.5, 6.25, and 3.125 mg/mL was mixed with 100 *μ*L overnight culture of bacteria in either 0.5 mL phosphate buffer, (−)S9 group, or 0.5 mL S9 mix, (+)S9 group. The composition of S9 mix was 5% v/v Aroclor-1253-induced rat liver S9 (MOLTOX, Molecular Toxicology Inc., Boone, NC, USA), 8 mM MgCl_2_, 33 mM KCl, 5 mM glucose-6-phosphate, 2 mM NADP, 0.1 M phosphate buffer, and pH 7.4. The mixture was subsequently mixed with agar solution containing histidine/biotin (Sigma-Aldrich, St. Louis, MO, USA) and being kept at 50 ± 1°C before transferring to minimal glucose agar plates. Solidified agar plates were incubated at 35 ± 1°C in the incubator for 48 ± 1 hours before counting colonies.

Distilled water was applied as a negative control, while for positive controls, chemical substances and corresponding concentrations applied in tests were summarized in Table 1.

### 2.3. *In Vitro* Chromosomal Aberration Test

The test was performed following OECD guidelines [10]. Chinese hamster ovary cells CHO-K1 were obtained from Bioresource Collection and Research Center (Hsinchu, Taiwan) and cultured in Ham F-12 medium supplemented with 10% fetal bovine serum in 37°C and 5% CO_2_ incubator. The test substance was dissolved in culture media containing 0.1% DMSO (v/v) in 5 serial dilutions: 5, 2.5, 1.25, 0.625, and 0.3125 mg/mL. The negative control was 0.1% DMSO (v/v) in culture media, and the positive controls were 2 *μ*M mitomycin C (Sigma-Aldrich, St. Louis, MO, USA) for (−)S9 group and 80 *μ*M cyclophosphamide monohydrate (Sigma-Aldrich, St. Louis, MO, USA) for (+) S9 group.

The test substance or controls were administered in three conditions. For short-term treatment, the substances were applied for 3 hours followed by a recovery period of 17 hours. For metabolic activation, the substances were applied together with S9 mix for 3 hours. For continuous treatment, the substances were kept in culture for 20-hours. At 20 hour posttreatment, cell viability was determined by MTT assay and specimen for chromosome observation was prepared in parallel experiments. In brief, 0.1 *μ*g/mL Colcemid solution (KaryoMAX Colcemid Solution, Gibco, Life technologies, Carlsbad, CA, USA) was added into the culture and incubated for 4 hours. Cells were harvested by trypsinization and centrifugation, swollen by freshly prepared 0.56% KCl, and fixed with ice-cold freshly prepared mixture of 3 : 1 methanol : glacial acetic acid. The smear was allowed to be air-dried and stained with Diff-Quik (Sysmex Corporation, Kobe, Japan) before microscopic observation.

The frequency of the cells with chromosome structural aberrations or numerical disorders was scored in 100 well-spread metaphases for each dose in duplicate. The aberrations were classified into 8 groups: chromosome gap (G), chromosome break (B), dicentric (D), ring (R), chromatid gap (g), chromatid break (b), multiple aberrations (MA), and acentric fragment (AF).

### 2.4. Animals

ICR male mice obtained from BioLasco (Taipei, Taiwan) were housed in cages (5 mice/cage) in the animal experiment room (National Yang-Ming University, Taipei, Taiwan). The temperature was set at 22 ± 3°C, humidity 40~70%, ventilation frequency 15 ± 5 times per hour, and lighting 12 hours per day. All mice had free access to water and the feed LabDiet 5010 Rodent Diet (Purina Mills LLC, St. Louis, MO, USA). The bedding was Aspen Chip (Northeastern Products Corp., USA).

### 2.5. Micronucleus Tests

The test was performed following OECD guidelines [11]. The negative control, reverse osmosis (RO) water, and test substance were administered 20 mL/kg at doses of 1.25, 2.5, and 5.0 g/kg by stainless feeding needles. The positive control cyclophosphamide (Sigma-Aldrich, St. Louis, MO, USA) was applied 10 mL/kg at the dose of 0.05 g/kg by intraperitoneal injection. The mice were observed daily for any posttreatment clinical symptoms, and their body weight was taken before treatment and 72 hours after treatment. At 48- and 72-hour posttreatment, 3-4 *μ*L blood was collected from tail vein and smeared on a microscope slide coated with acridine orange (Sigma-Aldrich, St. Louis, MO, USA). The smear sample was incubated at room temperature for 3 to 4 hours before fluorescent microscope observation. The frequency of reticulocytes (orange-red signal) was scored in 1000 red blood cells, while micronucleated reticulocytes (yellow-green signal) were scored in 1000 reticulocytes.

### 2.6. Statistics

All data obtained in this study were expressed in mean ± SD. The body weight and frequency of reticulocytes or micronucleated reticulocytes were subjected to one-way ANOVA and Duncan's multiple range tests by SPSS software (IBM, NY, USA). Significance of difference between groups was determined by *P* < 0.05.

## 3. Results and Discussion

### 3.1. Reverse Mutation Assay

We firstly validated the genotypes of test strains, including histidine requirement, rfa mutation, uvrB mutation, and ampicillin resistance (Table 2). TA97, TA98, and TA100 possessed all characteristics, while TA102 had no mutation on uvrB and TA1535 contained no plasmid that rendered ampicillin resistance, which were all consistent with the previous report [12, 13].

**Figure 1:**
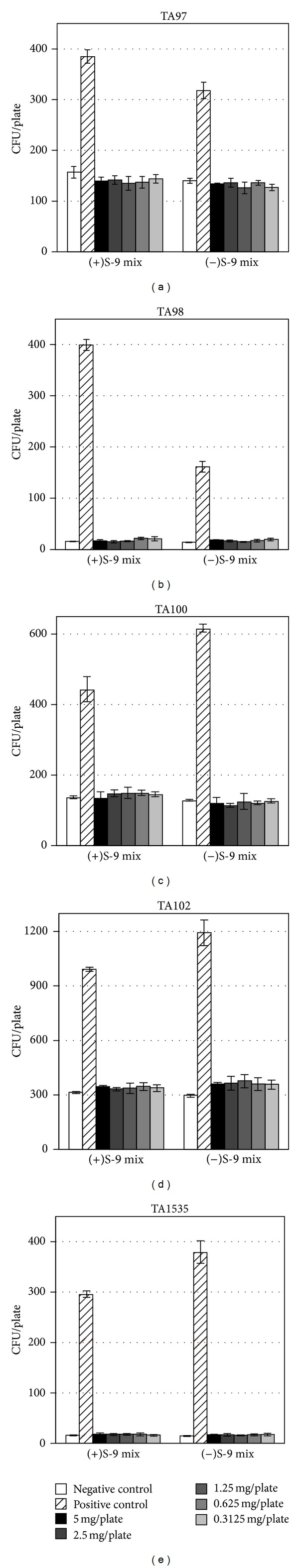
The multispecies probiotic mixture does not induce reverse mutation of *Salmonella* Typhimurium strains at dose levels between 0.3125 and 5 mg/plate. Metabolic activation of test substance is achieved by adding S9 mix. The graphs present the number of reverse mutated colonies grown on minimal agar plates (mean ± SD, *n* = 3).

Since our initial test revealed no pronounced toxicity on test strains at concentration as high as 5 mg/plate (data not shown), we set this concentration as the highest dose and performed the Ames test with its serial dilutions. As shown in Figure 1, compared to negative control groups (white bars), all positive control substances (hatched bars) induced at least 2-fold increase of the number of reverse mutation colonies, validating the effectiveness of the test. Moreover, we found that neither the test substance induced greater than 2-fold increase of reverse mutation at dose levels between 0.3125 and 5 mg/plate nor did the metabolically activated test substance (with S9 mix) exhibit mutagenicity for test strains. Taken together, our data suggest that the test substance does not induce bacterial reverse mutation in the current test conditions.

### 3.2. *In Vitro* Mammalian Chromosome Aberration Test

**Figure 2:**
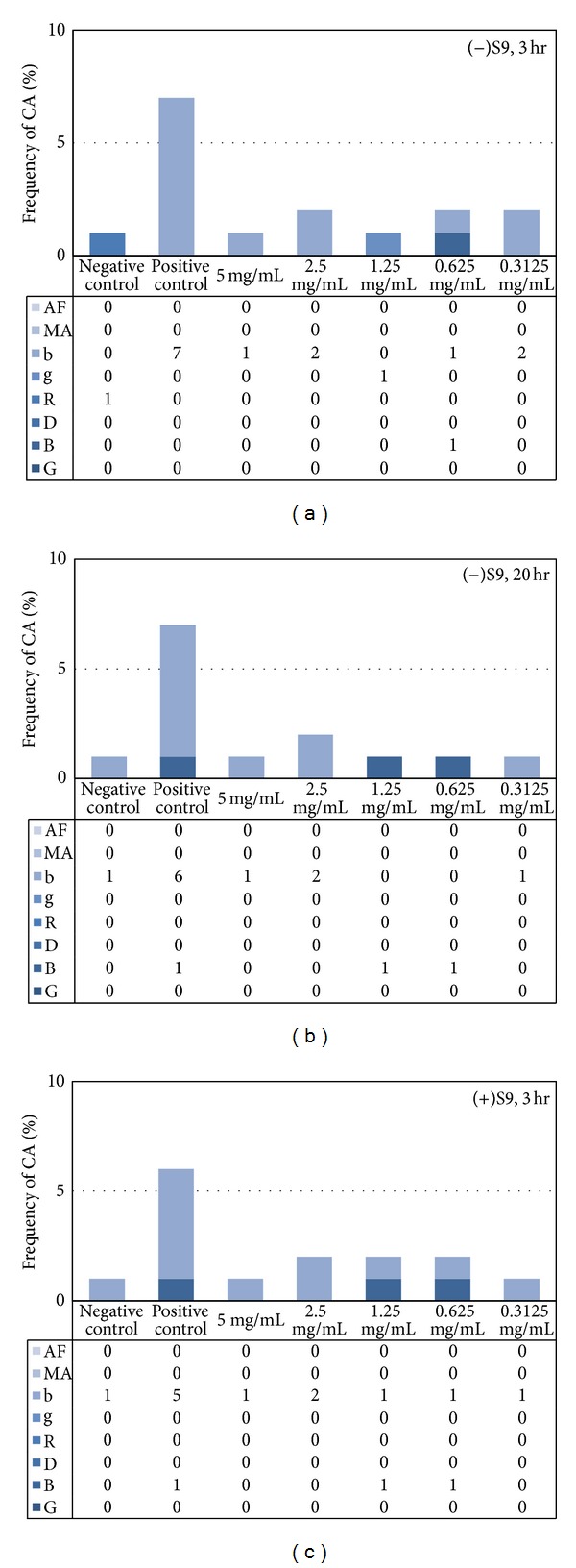
The multispecies probiotic mixture does not provoke the frequency of chromosomal aberration (CA) in mammalian cell culture. (a) Three hour exposure to test substances followed by 17-hour recovery period. (b) Continuous 20-hour exposure to test substance. (c) Metabolic activation of test substance by cotreatment with S9 mix for 3-hours followed by 17-hour recovery period. AF: acentric fragment; MA: multiple aberrations; b: chromatid break; g: chromatid gap; R: ring; D: dicentric; B: chromosome break; G: chromosome gap.

According to our initial test (data not shown), the test substance neither inhibited cell growth nor killed CHO cells, so we decided to set 5 mg/mL as the highest exposure level and use its serial dilutions for further dose-response tests. The negative control induced less than 3% cells with chromosomal aberrations, and positive control substance induced significant increase of aberrations (*P* < 0.01), providing validity of the tests (Figure 2). Neither short-term (3 hr) nor continuous (20 hr) treatment induced higher frequency of aberrations that were significantly different from negative controls. Likewise, metabolic activation of the test substance did not interfere with the mitotic process or cell cycle progression. In summary, these data indicate that exposure to the test substance does not result in chromosome aberrations in cultured mammalian somatic cells under the test conditions.

### 3.3. Micronucleus Assay with Mouse Peripheral Blood

**Figure 3:**
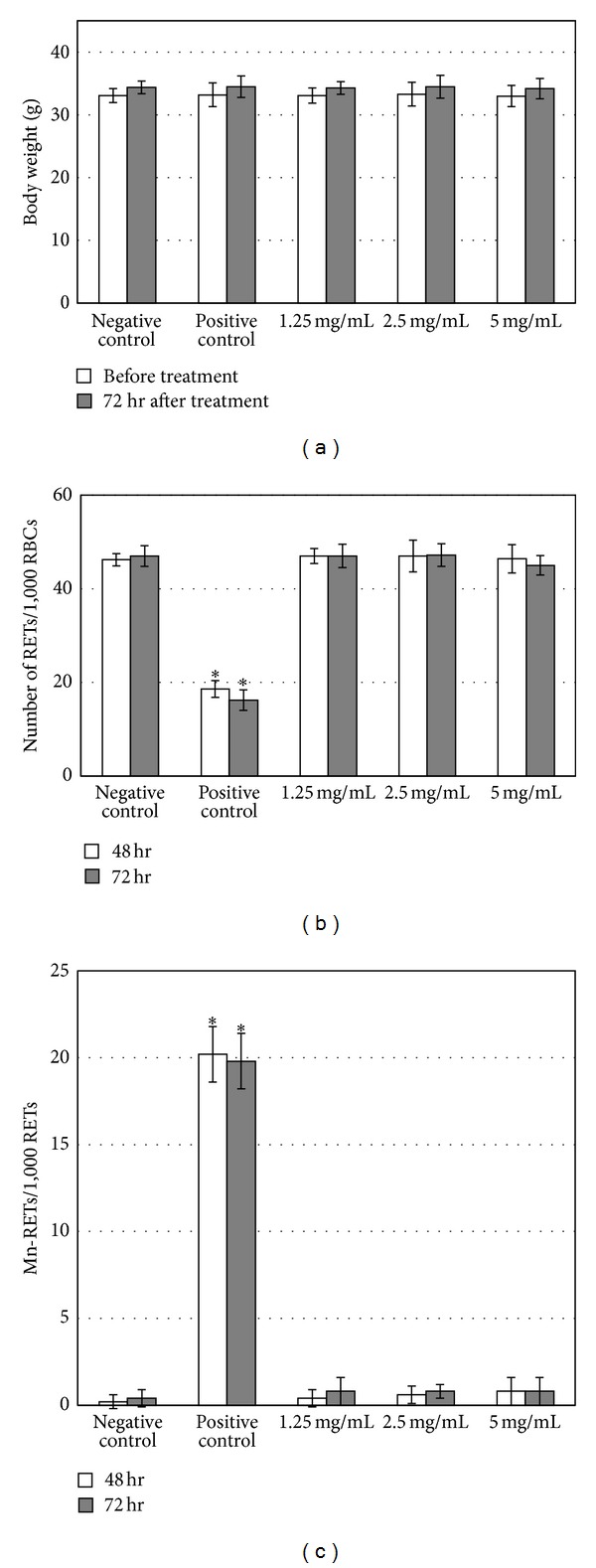
The multispecies probiotic mixture does not induce increased number of micronucleated reticulocytes in mouse peripheral blood. (a) Body weight of mice before and 72 hours after ingestion of the test substance. (b) The number of reticulocytes in 1000 red blood cells from the peripheral blood of mice treated with control and test substance. (c) The number of micronucleated reticulocytes in 1000 reticulocytes. All graphs present mean ± SD (*n* = 5). RETs: reticulocytes; RBCs: red blood cells; Mn-RETs: micronucleated reticulocytes.

We further investigated whether uptake of the multispecies probiotic mixture resulted in chromosome damages in mice by *in vivo* micronucleus tests. Test animals were grouped to receive treatments of negative control (RO water), positive control (0.05 g/kg cyclophosphamide), and the test substance in dose levels 1.25, 2.5, and 5 g/kg. During the 72-hour posttreatment, all mice exhibited no clinical symptoms and all gained some body weight after intakes of control and test substances (Figure 3(a)). We collected peripheral blood at 48- and 72-hour posttreatment and observed the frequency of reticulocytes and micronucleated reticulocytes (Figures 3(b) and 3(c), resp.). At 48-hour posttreatment, the rate of reticulocytes occurrence was 46.2 ± 1.3‰ in negative control group, but the rate decreased significantly (*P* < 0.05) to 18.6 ± 1.8‰ by cyclophosphamide treatment, suggesting that the chemotherapy drug has effectively induced myelosuppression (i.e., bone marrow suppression) [14]. High, medium, and low dose of the test substance did not change the abundance of reticulocytes significantly and each gave rise to 46.4 ± 3.0‰, 47.0 ± 3.4‰, and 47.0 ± 1.6‰ of reticulocyte frequency, respectively (Figure 3(b)). On the other hand, the micronucleated reticulocytes occurred at a rate of 0.2 ± 0.4‰ with negative controls, while cyclophosphamide provoked the rate to 20.2 ± 1.6‰  (*P* < 0.05), indicating substantial DNA damages during the cell cycle. The test substance did not lead to increased rate of micronucleated reticulocytes (Figure 3(c)). Likewise, the observations made at 72-hour posttreatment were all consistent with results from 48-hour posttreatment (Figures 3(b) and 3(c)). Based on these observations, we conclude that the multispecies probiotic mixture ingestion does not lead to chromosomal damages during cell cycle processes.

In the present study, the multispecies probiotic mixture showed no mutagenic potential that leads to bacteria reverse mutation, *in vitro* chromosome aberration, and micronucleated reticulocytes in mouse peripheral blood. The species included in the formula, *L. rhamnosus*, *B. adolescentis*, and *P. acidilactici*, are either natural inhabitants in human guts or extensively used in food processing through human history, so the safety of these microbes has not been widely questioned. However, whether a mixture of nonpathogenic species induces interspecies interactions and gives rise to toxicity is unknown. The current study indicates that the probiotic formula carries no detectable genotoxicity, providing supporting evidence for human experiences as well as for previous reports on the safety of these microbes [15–20]. 

The novel strains contained in the multispecies probiotic mixture, *L. rhamnosus* LCR177 and *P. acidilactici* PA318, were isolated from pickled vegetables and human feces, respectively. Along with *B. adolescentis* BA286, these strains are selected on the basis of their efficacy in lowering blood lipid and maintaining beneficial microflora in guts. Since the complex of probiotics may serve as dietary supplements that promote human health, its safety is required to be assessed. Our results have provided the safety profile of the multispecies probiotic mixture in aspects of mutagenicity in a dose level as high as 5 g/kg in mice, which is nearly 67-fold more than the recommended daily value for human beings (4.5 g/60 kg per day). Therefore, we conclude that consumption of the probiotic mixture is safe in the aspect of genotoxicity.

## Figures and Tables

**Table 1:** Chemical substances and concentrations used as positive controls for reverse mutation assay.

Strain	(−) S9 mix		(+) S9 mix	
	Chemical substance	Conc. (*μ*g/plate)	Chemical substance	Conc. (*μ*g/plate)
TA97	4-Nitroquinoline-N-oxide	0.5	2-Aminofluorene	4.0
TA98	4-Nitroquinoline-N-oxide	0.5	Benzo[a]pyrene	4.0
TA100	Sodium azide	0.4	2-Aminofluorene	4.0
TA102	Mitomycin C	0.5	Benzo[a]pyrene	4.0
TA1535	Sodium azide	0.4	2-Aminoanthracene	4.0

**Table 2:** Genotyping of the test bacterial strains.

Strains	Histidine requirement	ΔuvrB mutation	rfa mutation	Ampicillin resistance
TA97	+	+	+	+
TA98	+	+	+	+
TA100	+	+	+	+
TA102	+	Θ	+	+
TA1535	+	+	+	Θ

+: the strain carries the mutation; Θ: the strain does not possess the feature.
